# Effects of the New Lift-Thrust Operation in Laser Acupuncture Investigated by Thermal Imaging

**DOI:** 10.1155/2019/4212079

**Published:** 2019-12-09

**Authors:** Kun-Chan Lan, Chih-Yu Wang, Che-Chang Kuo, Shu-Chen Chang, Hsi-An Lin, Xin-Yu Wu, Jia-Yu Ding, Gerhard Litscher

**Affiliations:** ^1^Department of CSIE, National Cheng Kung University, 701 Tainan, Taiwan; ^2^School of Chinese Medicine, China Medical University, 404 Taichung, Taiwan; ^3^Department of Biomedical Engineering, I-Shou University, 824 Kaohsiung, Taiwan; ^4^School of Chinese Medicine for Post Baccalaureate, I-Shou University, 824 Kaohsiung, Taiwan; ^5^New Age Chinese Medicine and Healthcare Clinic, 807 Kaohsiung, Taiwan; ^6^Department of Hospitality Management, Tajen University, 907 Pingtung, Taiwan; ^7^Research Unit of Biomedical Engineering in Anesthesia and Intensive Care Medicine, Research Unit for Complementary and Integrative Laser Medicine, and TCM Research Center Graz, Medical University of Graz, Auenbruggerplatz 39, 8036 Graz, Austria

## Abstract

Acupuncture manipulation is one of the key factors affecting the performance of acupuncture in Traditional Chinese Medicine. Lift-thrust and twirl-twist are two of the most commonly used manipulation methods for needle acupuncture. We previously have developed a novel laser acupuncture model that emulates lift-thrust operation. In this study, we intend to show the effectiveness of such a model by applying it on the Neiguan acupoint (PC6). Stimulation was reported to be beneficial for improving cardiac output and peripheral circulation. Therefore, we hypothesized that the stimulation of laser acupuncture may increase the temperature of the subjects' fingertip due to increased peripheral blood flow. A thermal imager was used to measure the temperature change of subjects' fingertips. Through regression analysis, it has been shown that while PC6 is stimulated, laser acupuncture with lift-thrust operation caused a more rapid, stable, and lasting temperature rise of fingertip than that without lift-thrust operation. On the contrary, we observe no significant temperature change when a sham point nearby PC6 (a point which is not traditionally defined as the acupoint) was stimulated. Our results suggest the emulation of manipulation methods such as the lift-thrust operation could be a potential direction for the future development of laser acupuncture.

## 1. Introduction

Acupuncture is one of the most effective methods for Traditional Chinese Medicine (TCM) treatment, and its efficacy has been confirmed by many studies [1–6]. Acupuncture is a rapid, convenient, economical, and effective treatment; however, one reason for the limited popularity of acupuncture is patients' fear of pain, especially for anxious people and children. On the contrary, due to the advantage of painlessness and noninvasiveness, more and more researchers and clinicians are starting to consider the use of “laser acupuncture” for research and treatment in recent years.

Acupuncture manipulation is one of the key factors affecting the performance of acupuncture in Traditional Chinese Medicine [7]. A correct acupuncture manipulation is the indispensable process to obtain effective therapeutic effect when performing traditional acupuncture. Lift-thrust and twirl-twist are two of the most idiomatic acupuncture manipulation methods, which are the basic operations for performing reinforcement and reduction of “qi” in Chinese medicine. Lift-thrust is to move forward and backward, while twirl-twist is to twirl or twist clockwise and counterclockwise, when the needle achieves a certain depth in the acupuncture point [8]. One can perform “reinforcement” of qi by thrusting heavily and lifting lightly or perform “reduction” by thrusting lightly and lifting heavily. This is a way to induce and speed up the reaction of the so called de-qi.

Some previous studies have proposed to achieve the effect of lift-thrust and twirl-twist based on some optical principles, in which they suggested to change the power of laser acupuncture output to simulate the acupuncture manipulation [9–11]. Specifically, they implemented “lifting” operation by decreasing laser power and “thrusting” operation by increasing laser power. Based on this hypothesis, they also developed various algorithms to simulate “reinforcement” and “reduction”. Huang et al. further designed a portable laser acupuncture instrument with the reinforcement and reduction functions based on the same principle [12].

**Figure 1:**
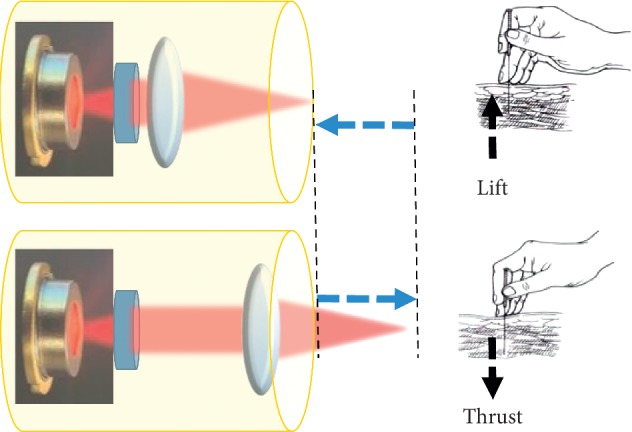
Schematic diagram of implementation of lift-thrust manipulating in laser acupuncture.

In our previous work, laser acupuncture instruments with the lift-thrust function have been developed [13, 14]. In the study, the lift-thrust function of laser acupuncture is implemented by moving the focused laser spot back and forth. The energy of the laser is concentrated at the focused light spot, which is considered as the tip of an acupuncture needle. When the laser light enters the human body, the position of the focused light spot is mobile as if the needle tip is moving through the acupuncture point, thereby realizing the lift-thrust function of the laser acupuncture.  Figure 1 shows a schematic diagram of how lift-thrust operation is simulated.

Within the current study, we used our previously developed emulated laser acupuncture with lift-thrust operation [13, 14] to investigate how it would affect possible changes of skin temperature. In the experiment, the laser acupuncture instrument was used to stimulate the Neiguan (PC6) point of the subjects for five minutes, and the thermal images before and after the laser acupuncture stimulation were taken with a thermal imager. Since it was reported that stimulating Neiguan (PC6) points would achieve the effect of relaxing blood vessels, improving cardiac output, and promoting the peripheral blood circulation [15], we expect it would increase the temperature of the subject's fingertips due to increased peripheral blood flow. The difference between with and without lift-thrust operation in the ability to improve the peripheral circulation should be verified.

## 2. Materials and Methods

### 2.1. Emulated Laser Acupuncture

**Figure 2:**
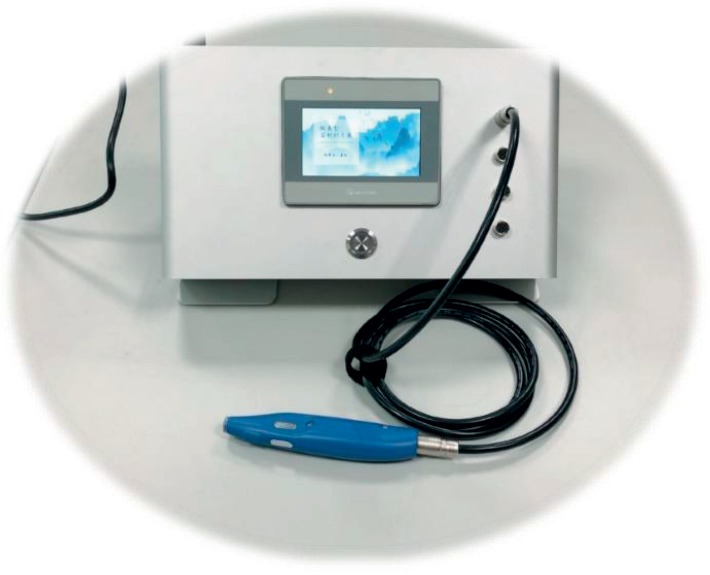
Prototype of the self-developed emulated laser acupuncture system.

In this study, a self-developed emulated laser acupuncture system (ELAS) with a lift-thrust function is used (Figure 2). This acupuncture instrument uses a Single-Board RIO (SBC, National Instruments, USA) as the core controller, which is programmed in LabVIEW. A touch panel is used as the input interface of the manipulation to setup the acupuncture depth (position of focused light spot), acupuncture force (light intensity), acupuncture techniques (reinforcing and reducing), acupuncture frequency, etc. The lift-thrust function was implemented by using a laser with focused light spot, which can be moved up and down by a mobile lens to emulate the traditional acupuncture manipulation [16]. The focused light spot moves in a range of 2 cm with a frequency of 0.5 Hz. A laser acupuncture mode without lift-thrust operation is implemented by fixing the focused light spot at 1 cm from the exit. The output power is set to 37.7 mW (which is much lower than the most commercially available laser acupuncture instrument). Lower power indicated much safer laser acupuncture treatment.

### 2.2. Thermal Imager

**Figure 3:**
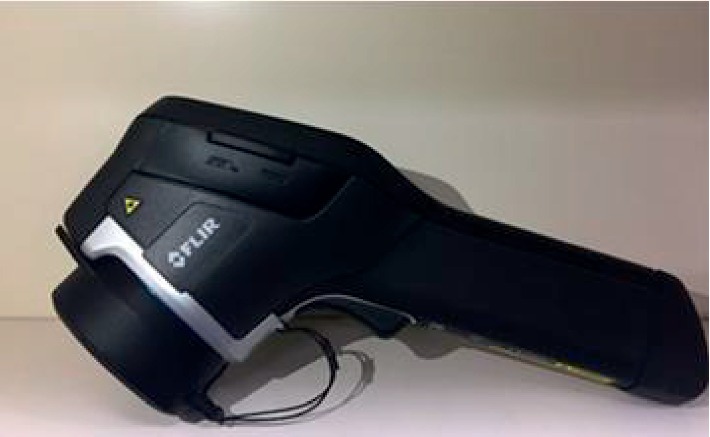
Thermal imager (E60, FLIR® Systems Inc., Wilsonville, OR, USA).

In this study, a thermal imager (E60, FLIR® Systems, Inc., Wilsonville, OR, USA) is used to record thermal images for evaluating the effect of laser acupuncture (Figure 3). Human body is a natural source of infrared radiation. Normally, the human body temperature is about 37°C, the skin temperature is about 28°C∼34°C, and its emissivity is 0.99. The thermal imager, which possesses a resolution of 320 × 240 and MSX® multispectral motion image enhancement technology for high-resolution thermal imaging, has the characteristics of high sensitivity and can detect the intensity of infrared light eradicating from the surface of human body. The original FLIR Tools® software, which can display temperatures in various colors and select the points for temperature measurement, is used for evaluation of skin temperature changes on the human body.

### 2.3. Selected Acupuncture Point (Neiguan, PC6)

**Figure 4:**
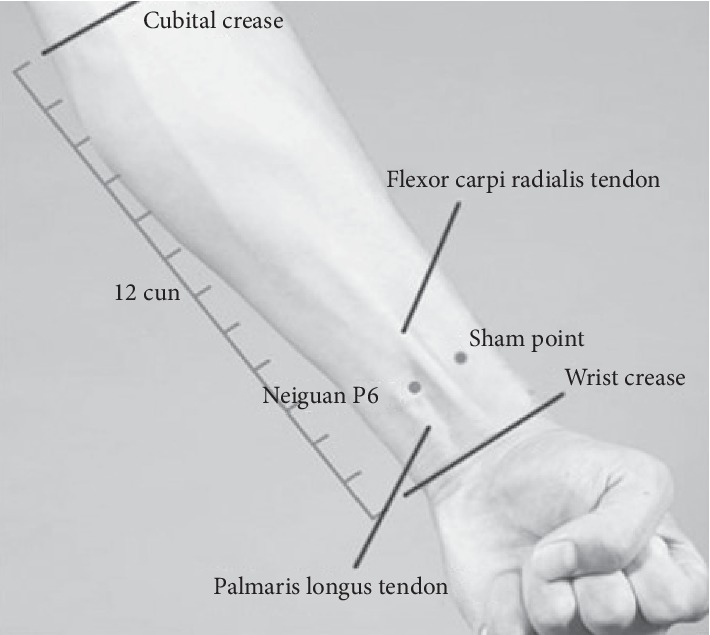
Position of the Neiguan point (PC6, belonging to pericardium meridian).

In this study, the Neiguan point (PC6, belonging to pericardium meridian) on the right arm was selected for our experiments. The Neiguan point is located at the volar side of the forearm, 2 cun above the wrist crease and between the longus tendon and the radial flexor tendon [17] (Figure 4). According to the literature, stimulating Neiguan can sedate nerves, regulate qi, and relieve pain. Modern research also confirmed that stimulating Neiguan can strengthen myocardial contractility and improve cardiac output and other cardiac functions [18–21]. We hypothesize that stimulating Neiguan with an emulated laser acupuncture instrument may increase blood flow, improve the peripheral circulation, and accordingly increase the temperature of the hand. Based on this hypothesis and previous results [15], we setup a thermal imager to take the thermal images of hand, measure, and analyze their temperature changes.

### 2.4. Experiments

In this study, we enrolled 60 healthy adults, aged 18–22 years old (35 female; 25 male), as experimental subjects. They were divided into three groups: with lift-thrust operation (group A), without lift-thrust operation (group B), and acupuncture on the sham point (group C). Each group recruited 20 subjects. For group A, laser acupuncture with lift-thrust operation was applied on the Neiguan point for 5 minutes. For group B, laser acupuncture without lift-thrust operation was applied on the Neiguan point for 5 minutes. For group C, laser acupuncture with lift-thrust operation was applied on a sham point (as shown in Figure 4) for 5 minutes.

None of the volunteers took medication at the time of examination. In addition, none of the persons knowingly had any peripheral circulatory disturbance. All volunteers were informed about the procedure as long as it did not influence the study scheme, and they gave their consent. The study which includes only noninvasive measurement methods was approved by the local ethics committee of the E-Da Hospital, Kaohsiung, Taiwan.

**Figure 5:**
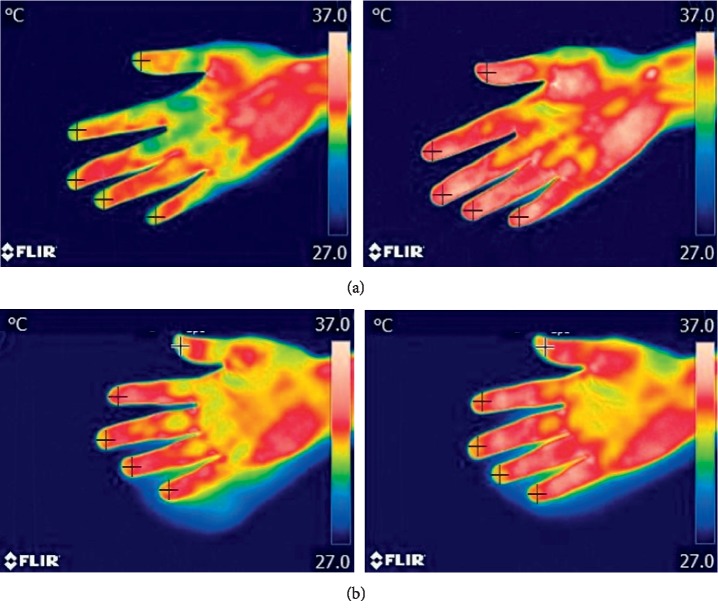
Thermal images of the palm before (left) and after (right) laser acupuncture: (a) with lift-thrust operation; (b) without lift-thrust operation.

**Figure 6:**
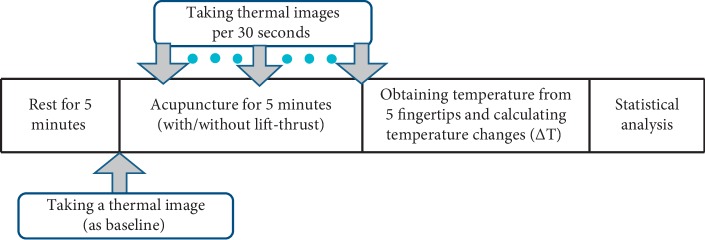
The experimental flow chart.

At first, the subjects took rest on a chair for 5 minutes to adapt to the room temperature and stabilize their breathing. Next, they were seated on the experimental chair, with the right arm placed straight on the experimental table. A thermal image of the palms and arms was taken before laser acupuncture application. Thermal images of the palms and arms were taken per 30 seconds until the end of the stimulating process. In addition, we examined the temperature change of fingertips based on the thermal image of the palm. As discussed previously, stimulating Neiguan points would achieve the effect of relaxing blood vessels, improving cardiac output, and promoting the peripheral blood circulation; thus, we expect it would increase the temperature of the subject's fingertips due to increased peripheral blood flow. For each thermal image, we recorded the temperature of five fingertips on the marked areas (denoted by “+” as shown in Figure 5(a)) and then averaged them. The temperature change (Δ*T*), defined as the temperature difference from the baseline, was then calculated for each individual subject.  Figure 6 shows the experimental flow chart.

### 2.5. Statistical Analysis

Since the data measured in the present study are not normally distributed, nonparametric statistical analyses were used in this study. The Wilcoxon signed-rank test was used to evaluate significant temperature differences between before and after laser acupuncture. The Mann–Whitney *U* test was used to evaluate significant temperature differences between laser acupuncture with and without lift-thrust operation. The difference is judged to be statistically significant if *P* < 0.05. Furthermore, a regression analysis with a quadratic fitting curve was used to present the temperature variation over time. The statistical software was SPSS Statistics 18.0.

## 3. Results

In this study we mainly conducted three kinds of analyses described as follows.

### 3.1. Temperature Differences between before and after Laser Acupuncture on Acupoint Neiguan

Figure 5 shows the temperature images of the palm before and after laser acupuncture for 5 minutes. Specifically, Figure 5(a) shows the thermal image before the laser acupuncture (left) and after the laser acupuncture (right) with lift-thrust operation.  Figure 5(b) shows the thermal image before the laser acupuncture (left) and after the laser acupuncture (right) without lift-thrust operation. It can be clearly seen that the temperature and the area changes of the palm are more obvious with lift-thrust operation than that without lift-thrust operation.

A One-sample Wilcoxon-signed rank test shows significant difference between before and after laser acupuncture application (*P* < 0.05), either with or without lift-thrust operation (Table 1).

### 3.2. Temperature Changes between Laser Acupuncture on Acupoint Neiguan with and without Lift-Thrust Operation over a 5-Minute Period

**Figure 7:**
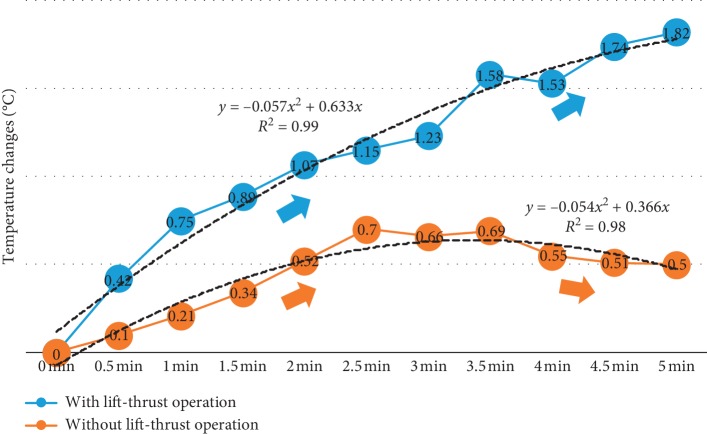
Linechart for temperature rise over time. Blue circle: laser acupuncture with lift-thrust operation. Orange circle: laser acupuncture without lift-thrust operation.

We conducted an experiment to evaluate the effect of laser acupuncture on the Neiguan point with and without lift-thrust operation. The temperature changes of subjects' fingertips were measured per 30 seconds.  Figure 7 shows the median of temperature changes ((Δ*T*) as defined in Section 2.4) of 20 subjects every 30 seconds during the laser acupuncture stimulation (the median, instead of the mean, is used here because our data are not normally distributed). As shown in Figure 7, laser acupuncture stimulation with lift-thrust operation results in a more rapid, stable, and lasting temperature rise. On the contrary, laser acupuncture stimulation without lift-thrust operation produces only a moderate temperature rise, reaching its highest temperature at about 2.5 minutes and then starting a slow decline. To further evaluate the time-dependent property, a quadratic regression analysis was used to show the trend of temperature changes over time. Equations (1) and (2) present the formula of fitting curves for laser acupuncture stimulation with and without lift-thrust operations, respectively:

(1) $$\begin{array}{c} \text{with lift}‐\text{thrust}:y=-0.057x^{2}+0.633x,\;\;\;R^{2}=0.99, \end{array}$$

(2) $$\begin{array}{c} \text{without lift}‐\text{thrust}:y=-0.054x^{2}+0.366x,\;\;R^{2}=0.98, \end{array}$$

where *y* is the temperature change from the baseline (°C) and *x* denotes time (in minutes). The parabolic curve with negative quadratic term indicates that the temperature rise will slow down with time. Given that the quadratic terms in (1) and (2) are not much different, and a larger linear term in (1) indicated a faster and sustained temperature rising for laser acupuncture with lift-thrust operation.

In terms of the significance of regression coefficients, the *R*^2^ values of both curves are higher than 0.98, indicating that the regression mode has a good explanatory power for the data. The *P* value of ANOVA analysis is less than 0.05, showing a significant regression relationship between the independent variable (time) and the dependent variable (temperature change). The *P* values of *t* statistic for all regression coefficients of both laser acupuncture with and without lift-thrust are smaller than 0.05, indicating that the regression coefficients are all significantly different from 0 (Table 2).

Table 3 shows an independent samples Mann–Whitney *U* test of the temperature difference between the laser acupuncture with and without lift-thrust function at the end of the stimulation. Result shows statistically significant difference between these two kinds of operation.

### 3.3. Temperature Differences between Laser Acupuncture with Lift-Thrust Operation on Acupoint and Sham Point

**Figure 8:**
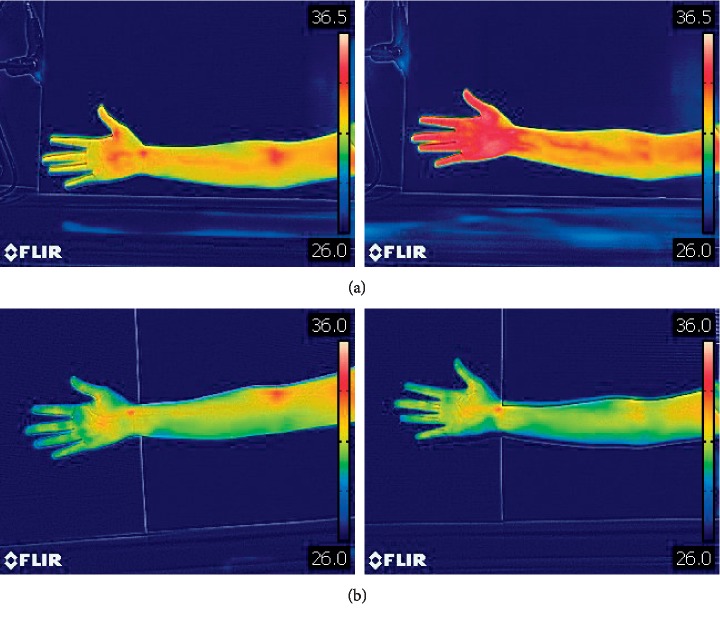
Thermal image of laser acupuncture stimulating the Neiguan point and the nonacupoint on the pericardium meridian for 5 minutes: (a) before laser acupuncture stimulation and after laser acupuncture stimulation; (b) before laser acupuncture stimulation and after laser acupuncture stimulation.

In order to understand whether temperature increase is due to the heating effect of laser light, we also applied laser acupuncture with lift-thrust operation on a sham point for 5 minutes and then took thermal images.  Figure 8 shows the thermal image of stimulating the Neiguan point and sham point for 5 minutes. Stimulation on the Neiguan point caused temperature rising of the hand, while stimulation on the sham point does not cause any obvious temperature change of the hand.

Table 4 shows the results of laser acupuncture stimulation on the Neiguan point and on the nonacupoint. It can be seen that the median temperature change between before and after laser acupuncture with lift-thrust operation on acupoint Neiguan was 1.82°C, while the temperature change between before and after laser acupuncture with lift-thrust operation on the sham point (a nonacupoint shown in Figure 4) was 0.25°C. The results of the One-sample Wilcoxon signed-rank test showed that there exists a significant difference between the temperature of before and after laser acupuncture with lift-thrust operation on Neiguan; while no significant difference between the temperature of before and after laser acupuncture with lift-thrust operation on the sham point.

In addition, Table 4 also shows a statistical result of laser acupuncture stimulating the acupoint/sham point. It is seen that no matter at the acupoint or sham point, there were no significant temperature changes at the location of the laser stimulation. Therefore, we believe that the temperature change caused by the laser stimulation on the Neiguan point is due to the physiological response and not resulted from the thermal effects of the interaction between the laser and the skin.

## 4. Discussion

Laser acupuncture is defined as “photonic stimulation of acupuncture points and areas to initiate therapeutic effects similar to that of needle acupuncture and related therapies together with the benefits of photo biomodulation (PBM)” [22]. The basics of laser acupuncture are well described in scientific literature [23–26].

In previous experiments we have clearly shown that basic acupuncture manipulations, lifting-thrusting and twisting-twirling, have influence on local acupoint skin temperature [27]. Compared with sham acupuncture, de-qi acupuncture can be able to increase the skin temperature of the stimulated acupoints. We have shown that the range of temperature increase caused by lifting-thrusting stimulation was higher than that of the twisting-twirling method.

Previous studies also showed that acupuncture manipulation through a steel needle can change physiological phenomena of the subject more effectively than laser stimulation [26]. Li et al. applied the lift-thrust operation to perform reinforcement and reduction manipulation and stimulate the Zusanli (ST36) acupoint of the subjects [28]. The results showed that the effect of the “reinforcement” manipulation caused significantly higher local blood perfusion than that of the “reduction” manipulation. Ping et al. used various reinforcement and reduction techniques to stimulate the Hegu (LI4) acupoint of the subject [29]. With a thermal imager, they found that increased temperature on the local skin around Hegu through reinforcement was significantly higher than that of the reduction manipulation. Chen et al. used a thermal imager to detect the temperature variation of acupoints on the same meridian [30]. More specifically, when the acupoint is stimulated, the temperature of the other acupoint on the same meridian might increase or decrease depending on the positions of acupoints.

In previous studies, we have already been able to prove that stimulation at acupuncture point Neiguan can lead to a temperature increase of up to about 2 degree Celsius at the fingertip [31]. At that time, these results could be achieved with a laser needle.

However, much research work is still waiting in the future. Thus, for example, it will be necessary to examine the different frequencies often used in laser acupuncture to see if it can bring about any further improvement in addition to the lift-thrust laser method described here.

Our current results described in this article are fully in line with the previous studies, and more importantly, the new lift-thrust operation technology for laser stimulation is expected to deliver much better clinical results in the future, which will translate into tremendous progress in the entire laser acupuncture field.

## 5. Conclusion

In this study, we carried out the verification of the effectiveness of the emulated laser acupuncture system and showed that the laser acupuncture with lift-thrust function resulted in significantly higher temperature increasing at the fingertip than that without lift-thrust function through stimulating the Neiguan point. Furthermore, laser acupuncture applied on the nonacupoint has no effect for increasing temperature even lift-thrust operation was used. This indicates that lift-thrust function is extremely helpful in improving the performance of the laser acupuncture. In addition, the laser power used in our system is relatively lower compared with some of the commercially available laser acupuncture instruments. Lower power indicates longer stimulating time, which is consistent with the practice of “needle retention” in traditional acupuncture. The innovative results of this research have created a novel laser acupuncture operation model, which provides a potential approach for the future development of laser acupuncture in accordance with the Traditional Chinese Medicine approach.

## Figures and Tables

**Table 1:** One-sample Wilcoxon signed-rank test before and after laser acupuncture stimulation.

Operation	Temperature difference	
	One-sample Wilcoxon signed-rank test	
	Δ*T* (after − before)	*P* value
With lift-thrust	1.82°C	<0.01^*∗∗*^
Without lift-thrust	0.5°C	0.018^*∗*^

^*∗*^ *P* < 0.05; ^*∗∗*^*P* < 0.01.

**Table 2:** Significance of regression coefficients.

	Coefficients	*P* value
(a) With lift-thrust		
Linear term (*β*_1_)	0.633	<0.01^*∗∗*^
Quadratic term (*β*_2_)	−0.057	<0.01^*∗∗*^

(b) Without lift-thrust		
Linear term (*β*_1_)	0.366	<0.01^*∗∗*^
Quadratic term (*β*_2_)	−0.054	<0.01^*∗∗*^

^*∗∗*^ *P* < 0.01.

**Table 3:** Independent samples Mann–Whitney *U* test for laser acupuncture operation with/without lift-thrust at the end of the stimulation.

Operation	Independent samples Mann–Whitney *U* test		*P* value
	With lift-thrust	Without lift-thrust	
Δ*T* (before and after)	1.82°C	0.5°C	<0.01^*∗∗*^

^*∗∗*^ *P* < 0.01.

**Table 4:** One-sample Wilcoxon signed-rank test for laser acupuncture stimulating the acupoint/sham point.

Operation	Temperature change			
	Fingertips		Laser-stimulating point	
	Δ*T* (before and after)	*P* value	Δ*T* (before and after)	*P* value
Acupoint	1.82°C	<0.01^*∗∗*^	0.06°C	0.37
Sham point	0.25°C	0.67	0.05°C	0.77

^*∗∗*^ *P* < 0.01.

## Data Availability

The original data used to support the findings of this study are available from the first author and from the corresponding author in Asia upon request.
